# Endoscopic tympanoplasty type I using interlay technique

**DOI:** 10.1186/s40463-022-00597-3

**Published:** 2022-11-17

**Authors:** Masahiro Takahashi, Masaomi Motegi, Kazuhisa Yamamoto, Yutaka Yamamoto, Hiromi Kojima

**Affiliations:** Department of Otorhinolaryngology, Jikei University School of Medicine, 3-25-8, Nishi-Shimbashi, Minato-ku, Tokyo 105-8461 Japan

**Keywords:** Transcanal endoscopic ear surgery, Middle ear surgery, Tympanic perforations, Myringoplasty, Interlay technique, Chronic otitis media

## Abstract

**Background:**

Tympanoplasty using the interlay technique has rarely been reported in transcanal endoscopic ear surgery, unlike the underlay technique. This is because many surgeons find it challenging to detach the epithelial layer of the tympanic membrane using only one hand. However, the epithelial layer can be easily detached from the inferior part of the tympanic membrane. Another key point is to actively improve anteroinferior visibility even if the overhang is slight because most perforations and postoperative reperforations are found in the anteroinferior quadrant of the tympanic membrane. We report the application of the interlay technique in endoscopic tympanoplasty type I for tympanic perforations.

**Methods:**

We retrospectively reviewed the medical records of 51 patients who had undergone tympanoplasty using the interlay technique without ossiculoplasty between 2017 and 2020. We then compared the data with those of patients who underwent microscopic surgery (MS) using the underlay technique between 1998 and 2009 (n = 104). No other technique was used in each group during this period. Repair of tympanic membrane perforation and hearing outcomes were assessed for > 1 year postoperatively.

**Results:**

The perforation sites were limited to the anterior, posterior, and anterior–posterior quadrants in 23, 1, and 27 ears, respectively. Perforations were closed in 50 of the 51 ears (98.0%), and the postoperative hearing was good (average air-bone [A-B] gap was 6.8 ± 5.8 dB). The surgical success rate for the repair of tympanic membrane perforation was not significantly different from the MS group (93.3%, *P* = 0.15). The average postoperative average A-B gap in the group that underwent the interlay technique was significantly different from that in the MS group (10.1 ± 6.6 dB, *P* < 0.01).

**Conclusion:**

The interlay technique should be considered as one of the treatment methods in endoscopic surgery for tympanic perforations. Further study of the postoperative outcomes of this procedure should be conducted to establish the optimal surgical procedure for tympanic perforations.

*Trial registration*: This study was retrospectively approved by the Institutional Review Board of the Jikei University, Tokyo, Japan (approval number: 32-205 10286).

**Video abstract:**

**Video abstract**

**Supplementary Information:**

The online version contains supplementary material available at 10.1186/s40463-022-00597-3.

## Background

Recent rapid improvements in endoscopic imaging technology have enabled transcanal endoscopic ear surgery (TEES) without microscope, and TEES has become a widely used approach to tympanic perforation [1–15]. Previous studies have reported the benefits of TEES, compared with those of a postauricular incision, which include decreased hospital stay [3], enhanced visualization of the operative field [4–6], operation time [8–11], and good cosmetic results [3, 5, 6]. These studies applied the underlay technique, although the grafts used varied from temporalis fascia, perichondrium, and cartilage. Tympanoplasty using the interlay technique, in which the graft is placed between the epithelial and the fibrous layers, has a high perforation closure rate due to graft stability [16, 17]; however, few studies have reported on this technique. Moreover, the interlay technique has rarely been reported in TEES [7]. This is because the underlay technique is relatively easier to perform using only one hand [18], and many surgeons find it challenging to detach the epithelial layer of the tympanic membrane (TM) in the interlay technique. In fact, considering the endoscopic advantages of close proximity and magnification, the interlay technique may be an option in endoscopic surgery. Most recently, the effectiveness of the interlay technique in TEES was reported [19]. However, the authors limited the patients to children with small perforations and did not report specific methods and tips. Therefore, we report the methods of endoscopic tympanoplasty type I for tympanic perforations using the interlay technique without limiting patients.

## Methods

We retrospectively reviewed the medical records of 51 patients (26 males and 25 females; mean age, 49.0 ± 23.5 years; range, 6–83 years) who underwent tympanoplasty using the interlay technique without ossiculoplasty for tympanic perforations in the Department of Otolaryngology at our institution, between August 2017 and July 2020. During this period, no surgery was performed except for the interlay technique. Ear canal width and perforation size were not exclusionary factors. We excluded patients with cholesteatomas and a history of ear surgery. Repair of TM perforation and hearing outcomes were assessed for > 1 year postoperatively based on recent reports. Hearing levels were the average of dB readings at 500, 1000, 2000, and 3000 Hz. We interpolated a 3000 Hz threshold by averaging the thresholds at 2000 Hz and 4000 Hz when 3000 Hz thresholds were not available, according to the guidelines [20].

Rigid endoscopes with angles of 0° or 30° (length: 18 cm, outer diameter: 2.7 mm; Storz, Germany) connected to a camera head (Storz, Germany) and a high-definition monitor were positioned in front of the surgeon. All surgeries were performed under general anesthesia as follows.

**Fig. 1:**
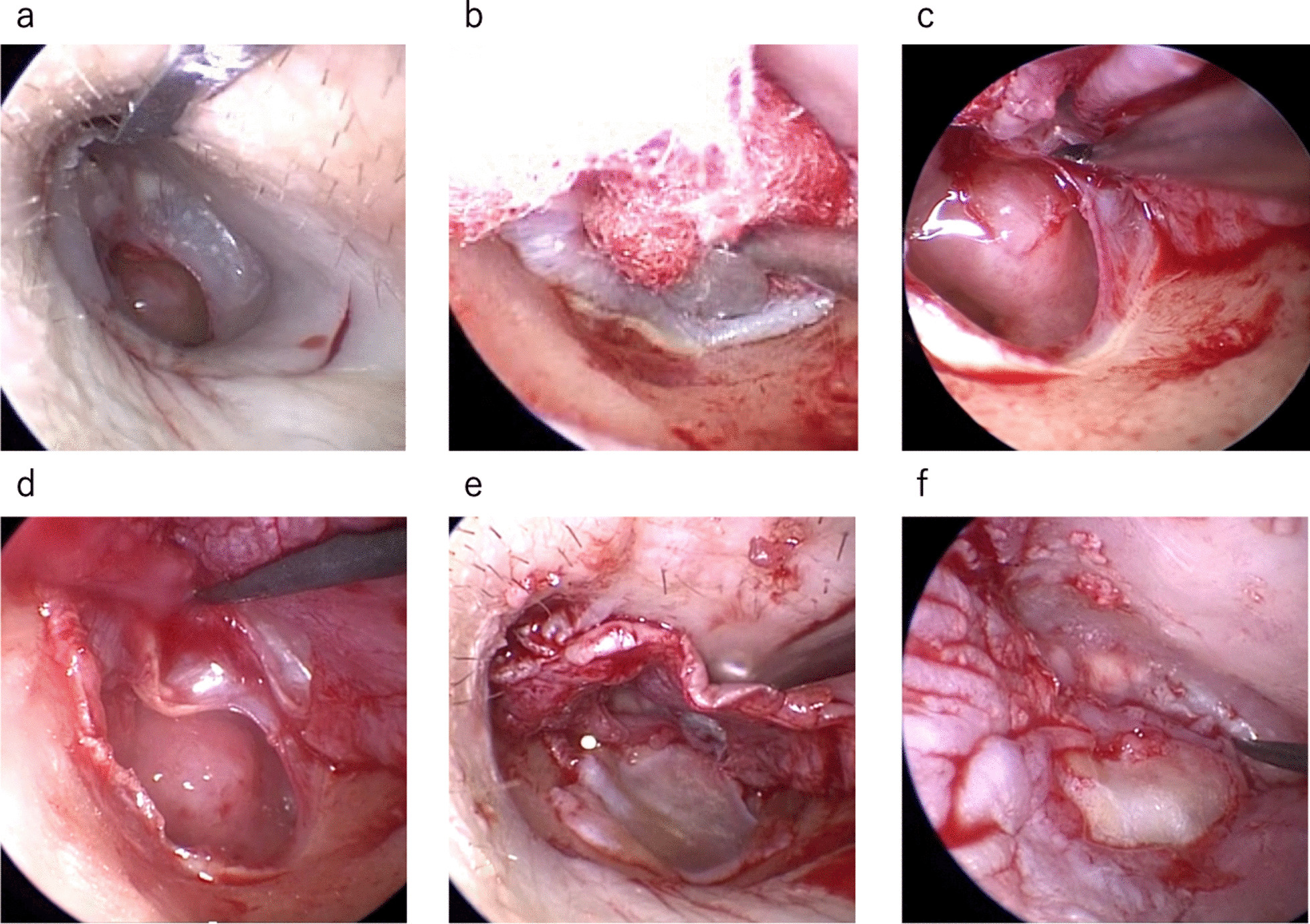
0° endoscopic image; A series of operations using the interlay technique. **a** The arched incision in the ear canal. **b** Identification of tympanic anulus. **c** Detaching the epithelial layer from the inferior part of the TM where the tympanic annulus was closely attached. **d** Detaching the epithelial layer surrounding the perforation. **e** The graft using the retroauricular fascia or tragus perichondrium was placed between the epithelial layer and the fibrous layer. **f** Confirmation of no gap between the reconstructed graft and the TM

**Fig. 2:**
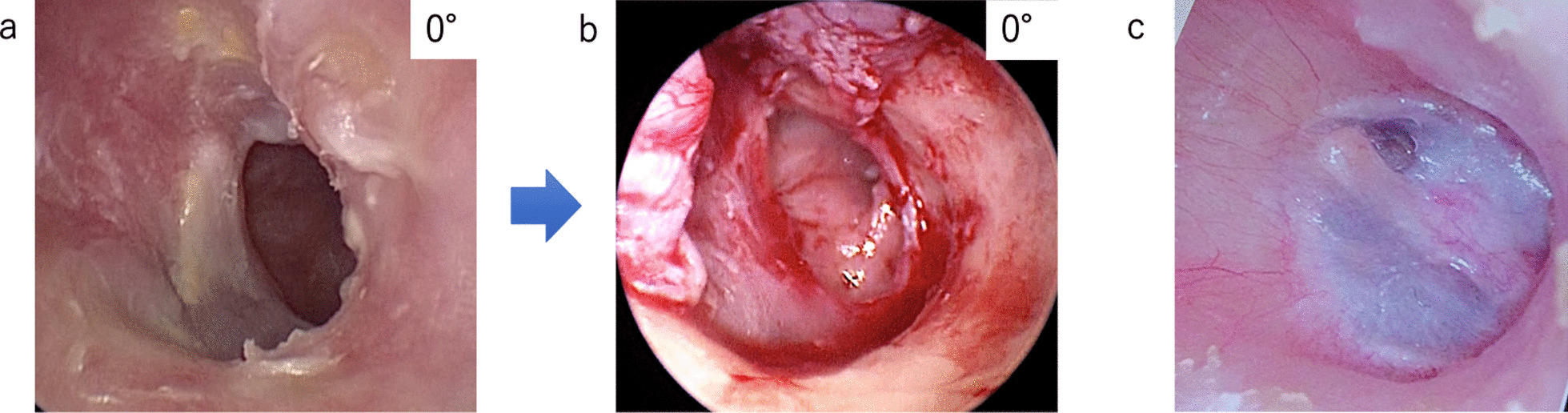
0° endoscopic image; a case with an overhanging anteroinferior wall. **a** Preoperative endoscopic image. The anteroinferior edge of the perforation is not visible. **b** Endoscopic image after shaving the anteroinferior wall. Note the good field of view that allows visualization of the entire perforation and instrumentation. **c** Endoscopic image 1 year postoperatively

1. Using arcuate incisions (Fig. 1a), a tympanomeatal flap was elevated from the posterior external auditory canal (EAC). The incision is made at approximately 210° downward, starting at approximately 1 cm to the lateral process of the malleus, which is more inferior than the approach to the attic or the stapes.
2. The tympanic annulus was identified (Fig. 1b). Then, the epithelial layer was first raided off the inferior TM as this is where the annulus is more firmly attached to the temporal bone (Fig. 1c). By starting the dissection of the epithelium inferiorly in this way, it is less likely that all layers of the TM are elevated inadvertently.
  *The edges of the perforation are freshened only when the epithelium is entrapped and transitions to a cholesteatoma.
3. The epithelial layer around the perforation was detached (Fig. 1d). It is important to avoid peeling the skin near the tympanic annulus on the anterior wall to prevent anterior blunting.
  *If perforation extends to the anterior tympanic annulus and there is no epithelial layer to detach, we detach the mucosa in the tympanic cavity to make space for placing the graft between the tympanic annulus and the mucosal layer.
  *Since detachment of the epithelial layer of the umbo is to be avoided as much as possible, a cut should be made in the graft to be placed in the anterior quadrant. If detachment is unavoidable, fibrin glue is used to completely adhere the raised epithelial layer to the umbo during repositioning.
4. The mobility of the ossicles was assessed by partially elevating all layers of the membrane or by using a 30° endoscope through the perforation.
5. Granulation or sclerosing lesions in the middle ear space were removed.
6. The harvested graft (temporalis fascia or tragal perichondrium) was placed in an interlay fashion by positioning it medial to the raised epithelium and lateral to the fibrous layer (Fig. 1e).
7. The tympanomeatal flap was repositioned at its original position. Care should be taken not to allow epithelial fragments to stray under the graft to avoid epidermal cholesteatoma.
8. The absence of any gap between the reconstructed tissue and the TM was confirmed (Fig. 1f).
9. The graft and the flap were fixed using fibrin glue. Absorbable gelatin (Gelfoam; Pfizer, USA) and a surgical sponge (Merocel; Medtronic, Dublin, Ireland) were packed into the EAC.
  * If the anteroinferior wall is overhanging (Fig. 2a), the bone is shaved with a curved bur (VISAO or MR8; Medtronic, Dublin, Ireland) and/or chisel and hammer endoscopically. The bone should be shaved until the anteroinferior edge of the perforation is visible, and the instrument can be easily operated (Fig. 2b). In addition, the dilation makes it easier to observe the eardrum postoperatively (Fig. 2c).

We then compared it to microscopic surgery (MS) using the underlay technique between 1998 and 2009 (n = 104; mean age, 43.3 ± 21.1 years). No other technique was used during this period, and the surgical indication criteria were the same as those for TEES with the interlay group. A single surgeon performed the procedure in each group. The results are expressed as means with 95% confidence intervals. All analyses were performed using a statistical software package (JMP version 13; JMP pro 14.0.0; SAS Institute Japan, Tokyo, Japan) and included the t-test, paired t-test, and Fisher’s exact probability test for assessments of differences between groups. A p-value of < 0.005 denoted the presence of a statistically significant difference. This study was approved by the Institutional Review Board of the Jikei University, Tokyo, Japan (approval number: 32–205 10,286).

## Results

In the TEES with interlay group, the size of the perforations for 1, 2, 3, and 4 quadrants were 9, 31, 8, and 3 ears, respectively. The perforation sites were limited to the anterior, posterior, and anterior–posterior quadrants in 23, 1, and 27 ears, respectively. The surgical success rate for TM closure was 98.0% (50/51 ears). The mean preoperative air conductive (AC) threshold was 43.5 ± 18.7 dB, and the air-bone (AB) gap was 19.2 ± 9.4 dB, while the postoperative AC threshold was 30.5 ± 17.5 dB, and the AB gap was 6.8 ± 5.8 dB. Both parameters were significantly improved (*P* < 0.01) (Table 1), and the AB gap in all cases was within 20 dB, which has been described as a successful hearing result 1 year postoperatively in previous reports [21, 22]. No cases showed anterior blunting and deterioration of bone conduction (BC) hearing (*P* = 0.86) (Table 1). Compared with the MS with underlay group, the rate of successful TM healing was not significantly different (*P* = 0.15), and the postoperative AB gap in the TEES with interlay group was significantly better (*P* < 0.01) (Table 1). Both groups were followed up for > 1 year, i.e., 2018 to 2021 and 1999 to 2010 for the TEES and MS groups, respectively.

**Table 1:** Characteristics of both groups and postoperative results

	Group		*P* value^a^
	TEES with interlay (n = 51)	MS with underlay (n = 104)	
Age (± SD) years	49.0 (± 23.5)	43.3 (± 21.1)	n.s
Size of perforations 1/2/3/4 quadrants (%)	9/31/8/3 (17.6/60.7/15.7/5.9)	27/43/23/11 (26.0/41.3/22.1/10.6)	
Sites of perforations AQ/PQ/both (%)	23/1/27 (45.1/2.0/52.9)	38/9/57 (36.5/8.7/54.8)	
Successful tympanic membrane healing	50 (98.0%)	96 (92.3%)	n.s
Sites of reperforations AQ/PQ/both	1/0/0	5/1/2	
PTA BC			
Preoperative (± SD) dB	24.2 (± 17.2)	16.0 (± 13.6)	*P* < 0.01
Postoperative (± SD) dB	23.6 (± 16.1)	15.9 (± 13.1)	*P* < 0.01
*P* value^a^	*P* = 0.86	*P* = 0.96	
PTA AC			
Preoperative (± SD) dB	43.5 (± 18.7)	36.6 (± 16.4)	*P* < 0.05
Postoperative (± SD) dB	30.5 (± 17.5)	26.0 (± 14.6)	n.s
*P* value^a^	*P* < 0.01	*P* < 0.01	
ABG			
Preoperative (± SD) dB	19.2 (± 9.4)	20.7 (± 8.8)	n.s
Postoperative (± SD) dB	6.8 (± 5.8)	10.1 (± 6.6)	*P* < 0.01
*P* value^a^	*P* < 0.01	*P* < 0.01	

Successful tympanic membrane healing and audiometric outcomes expressed as median (± SD) dB for transcanal endoscopic ear surgery (TEES) with interlay technique and microscopic surgery (MS) with underlay technique

AQ, only anterior quadrant; PQ, only posterior quadrant. ABG, air–bone gap; PTA BC, pure-tone average bone conduction; PTA AC, pure-tone average air conduction; n.s. , not significant

^a^*P* value < 0.05 is considered statistically significant

## Discussion

In a previous review of myringoplasty or endoscopic tympanoplasty type I [7], the perforation closure rate was 69–100% (mean, 88%), the mean postoperative AB gap was 4.0–18.1 dB (mean, 10.8 dB), and 77–100% (average, 90.8%) of the cases with a postoperative AB gap, were within 20 dB. That review was conducted with the underlay technique and had mostly limited target cases due to the sizes of the perforations and anatomical features. Therefore, we believe that our study, which did not limit the target cases, is a significant report, and the surgical success rate for TM closure and the postoperative hearing were good in patients who underwent the described surgical procedure. Comparing the two groups, the TEES with interlay group tended to have a higher perforation closure rate (92.3% vs. 98.0%) than the MS with underlay group, although there was no significant difference. The postoperative AB gap was significantly smaller (10.1 ± 6.6 vs. 6.8 ± 5.8 dB) in the TEES with interlay group. The significant difference in BC threshold (Table) may have resulted from the difference in the average patient age (43.3 vs. 49.0 years).

The interlay technique, in which the graft is placed between the epithelial and fibrous layers, is effective for graft stability [16, 17], but it has been rarely reported in TEES. This could be because most surgeons find detaching the epithelial layer of the TM using only one hand difficult. The epithelial layer can be easily detached from the inferior part of the TM, where the tympanic annulus is more closely attached. After confirming the layer to be detached, the epithelium should be detached parallel to the tympanic annulus using a round knife with no angulation. If the epithelium is detached perpendicular to it, the tympanic annulus is detached from the bone, making it difficult to detach without countertraction. Additionally, the ear canal skin near the anterior wall tympanic annulus should not be detached so as to prevent anterior blunting.

**Fig. 3:**
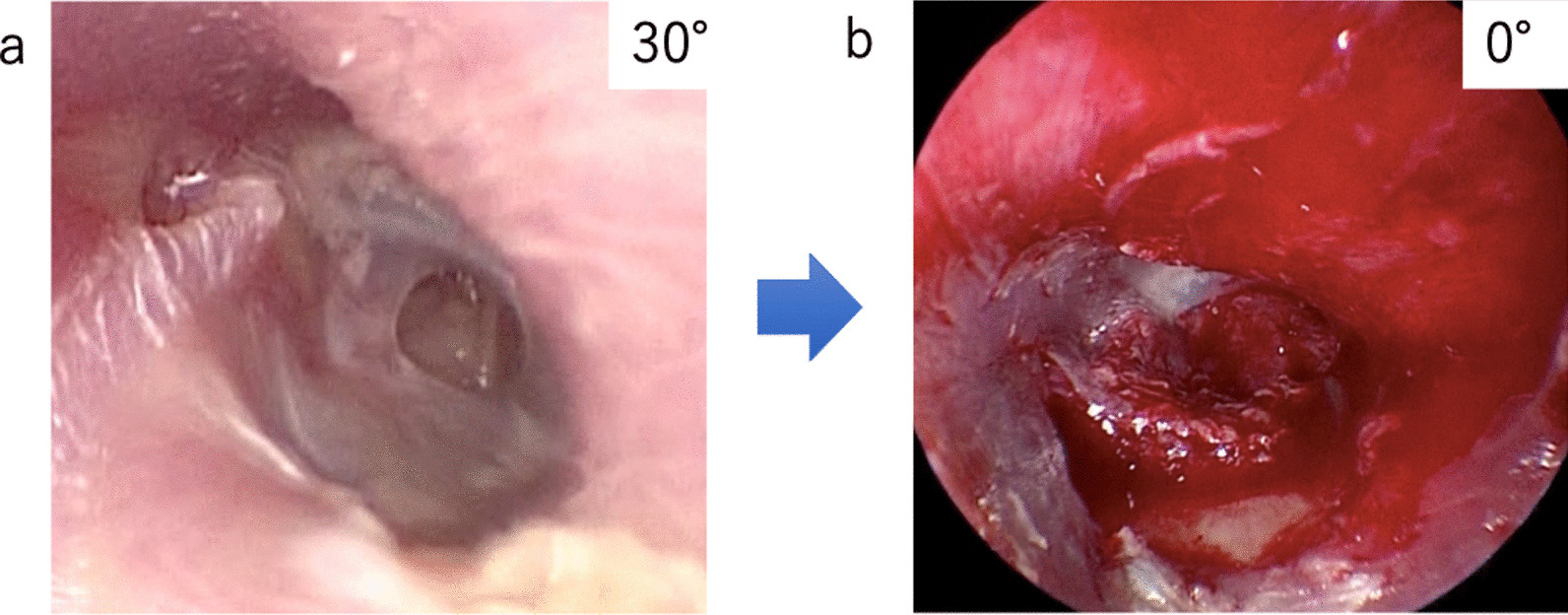
Endoscopic image; a case with slight overhanging of the anterior wall. **a** 30° endoscopic image before surgery. The field of view allows visualization of the entire perforation but does not facilitate ease of instrumentation. **b** 0° endoscopic image after myringoplasty without shaving the anterior wall. Note the damage to the anterior wall and poor visibility

Another key point is to actively improve anteroinferior visibility. The edge of anterior perforation is often visible in TEES without drilling overhang due to a wide field of view (Fig. 3a), even if the EAC is curved and the perforation is not fully visible in MS. However, the visual field for visual recognition and that for operation are different. The available field of view for visual recognition may be inadequate for a successful surgery. This is important because the epithelial layer is detached from the inferior part of the TM; additionally, most perforations and postoperative reperforations are found in the anteroinferior quadrant of the TM [23], and anterior perforation is also considered as a poor prognostic factor for graft uptake because of difficulty in access and graft instability [13, 14]. In other words, the cause of the high rate of anterior reperforation is presumably a technical problem. This is supported by the fact that the localization of stem cells is not different between the anterior and posterior quadrants [24]. Another reason for better anteroinferior visibility is that iatrogenic mechanical damage to the anterior wall may further worsen the surgical field (Fig. 3b), potentially resulting in an incomplete surgery. Moreover, a scab can develop on the ear canal postoperatively and hinder wound healing.

Other studies using cartilage on type-I endoscopic tympanoplasty reported a closure rate of 91.3–94.4% [11, 13, 15]. These findings indicate that the use of cartilage is a good choice in terms of graft stability. However, the perforation closure rate is still not 100%, and the TM becomes thicker when cartilage is used, making it difficult to determine if there is effusion. Therefore, we believe that it is preferable to use retroauricular fascia or tragus perichondrium. Moreover, these previous reports [11, 13–15] did not mention drilling of the anterior wall for good visualization. Although these reports stated that the field of view could be obtained by endoscopic surgery, we believe that in many cases, this is not sufficient. Concerning drilling EAC, we, alongside other institutions, have reported atticotomy or antrotomy in TEES using curved bur [25–27], and a similar technique can be performed for drilling anterior overhang.

Furthermore, several studies have recently reported on the effectiveness of Endoscopic Inlay Butterfly Cartilage Tympanoplasty [28–30], which does not require detachment of the TM and has a high perforation closure rate. However, indications for this procedure, with regard to the perforation size, are controversial [28, 29], and the procedure is not appropriate for the repair of intratympanic lesions. Interlay technique using an endoscope can be confirmed by analyzing the tympanic cavity, such as the ossicular chain, by temporarily peeling off a small part of the posterior TM in all layers or using a 30° endoscope through the perforation, which is difficult to achieve using the interlay technique in MS.

The limitations of our study are its retrospective nature and the relatively small sample size. In addition, a comparison of the underlay and interlay techniques in endoscopic surgery was not performed, and limited surgeons performed the surgery. Therefore, treatment efficacy could not be determined. A randomized controlled trial with four groups (microscopic interlay technique, microscopic underlay technique, endoscopic interlay technique, and endoscopic underlay technique groups) is required.

## Conclusions

Based on the postoperative results, the interlay technique should be considered as one of the treatment methods in endoscopic surgery for tympanic perforations.

## Data Availability

The datasets used and/or analyzed during the current study are available from the corresponding author on reasonable request.

## Abbreviations

TEES: Transcanal endoscopic ear surgery
TM: Tympanic membrane
EAC: External auditory canal
MS: Microscopic surgery
AC: Air conductive
BC: Bone conduction
AB: Air-bone

## References

[1] Han SY, Lee DY, Chung J, Kim YH (2019). Comparison of endoscopic and microscopic ear surgery in pediatric patients: a meta-analysis. Laryngoscope.

[2] Creighton FX, Kozin E, Rong A, Cohen M, Lee D (2019). Outcomes following transcanal endoscopic lateral graft tympanoplasty. Otol Neurotol.

[3] Tseng CC, Lai MT, Wu CC, Yuan SP, Ding YF (2017). Comparison of the efficacy of endoscopic tympanoplasty and microscopic tympanoplasty: a systematic review and meta-analysis. Laryngoscope.

[4] Anzola JF, Nogueira JF (2016). Endoscopic techniques in tympanoplasty. Otolaryngol Clin North Am.

[5] Miller KA, Fina M, Lee DJ (2019). Principles of pediatric endoscopic ear surgery. Otolaryngol Clin North Am.

[6] Nassif N, Berlucchi M, Redaelli de Zinis LO (2015). Tympanic membrane perforation in children: Endoscopic type I tympanoplasty, a newly technique, is it worthwhile?. Int J Pediatr Otorhinolaryngol.

[7] Ohki M, Kikuchi S, Tanaka S (2019). Endoscopic type 1 tympanoplasty in chronic otitis media: comparative study with a postauricular microscopic approach. Otolaryngol Head Neck Surg.

[8] Huang TY, Ho KY, Wang LF, Chien CY, Wang HM (2016). A comparative study of endoscopic and microscopic approach type 1 tympanoplasty for simple chronic otitis media. J Int Adv Otol.

[9] Choi N, Noh Y, Park W, Lee JJ, Yook S, Choi JE (2017). Comparison of endoscopic tympanoplasty to microscopic tympanoplasty. Clin Exp Otorhinolaryngol [Internet].

[10] Kaya I, Sezgin B, Sergin D, Ozturk A, Eraslan S, Gode S (2017). Endoscopic versus microscopic type 1 tympanoplasty in the same patients: a prospective randomized controlled trial. Eur Arch Otorhinolaryngol.

[11] Shakya D, Kc A, Tamang N (2021). Ajit Nepal Endoscopic versus microscopic type-I cartilage tympanoplasty for anterior perforation - a comparative study. Acta Otolaryngol.

[12] Tseng CC, Lai MT, Wu CC, Yuan SP, Ding YF (2016). Endoscopic transcanal myringoplasty for anterior perforations of the tympanic membrane. JAMA Otolaryngol Head Neck Surg.

[13] Mohanty S, Manimaran V, Umamaheswaran P, Jeyabalakrishnan S, Chelladurai S (2018). Endoscopic cartilage versus temporalis fascia grafting for anterior quadrant tympanic perforations—a prospective study in a tertiary care hospital. Auris Nasus Larynx.

[14] Marchioni D, Molteni G, Presutti L (2011). Endoscopic anatomy of the middle ear. Indian J Otolaryngol Head Neck Surg.

[15] Casas AL, Ruiz R, De Pauli D (2022). Endoscopic type 1 tympanoplasty; a composite graft technique for subtotal and total perforations. Eur Arch Otorhinolaryngol.

[16] Sheehy JL, Anderson RG (1980). Myringoplasty. A review of 472 cases. Ann Otol Rhinol Laryngol.

[17] Komune S, Wakizono S, Hisashi K, Uemura T (1992). Interlay method for myringoplasty. Auris Nasus Larynx.

[18] Harris JP, Wong YT, Yang TH, Miller M (2016). How I do it: Anterior pull-through tympanoplasty for anterior eardrum perforations. Acta Otolaryngol.

[19] Ranguis SC, Leonard CG, James AL (2021). Prospective comparison of pediatric endoscopic lateral graft and interlay tympanoplasty. Otol Neurotol.

[20] Gurgel RK, Jackler RK, Dobie RA, Popelka GR (2012). A new standardized format for reporting hearing outcome in clinical trials. Otolaryngol Head Neck Surg.

[21] Sakai M (1994). Proposal of a guideline in reporting hearing results in middle ear and mastoid surgery. Am J Otol.

[22] Eliades SJ, Limb CJ (2013). The role of mastoidectomy in outcomes following tympanic membrane repair: a review. Laryngoscope.

[23] Fermi M, Maccarrone F, Villari D, Palermo F, Alicandri-Ciufelli M, Ghirelli M (2021). Endoscopic tympanoplasty type I for tympanic perforations: analysis of prognostic factors. Eur Arch Otorhinolaryngol.

[24] Knutsson J, von Unge M, Rask-Andersen H (2011). Localization of progenitor/stem cells in the human tympanic membrane. Audiol Neurootol.

[25] Takahashi M, Yamamoto Y, Kojima H (2021). Transcanal endoscopic approach for pars flaccida cholesteatoma using a 70-degree angled endoscope. Eur Arch Otorhinolaryngol.

[26] Kakehata S, Watanabe T, Ito T, Kubota T, Furukawa T (2014). Extension of indications for transcanal endoscopic ear surgery using an ultrasonic bone curette for cholesteatomas. Otol Neurotol.

[27] Chen Y, Hu J, Liu W, Wang Q, Li Y, Peng A (2020). The treatment of cholesteatomas involving the antrum and mastoid using transcanal underwater endoscopic ear surgery. Otol Neurotol.

[28] Alian H, Esmat NH, Ohad H, Yona Y, Nageris BI (2016). Butterfly myringoplasty for total, subtotal and annular perforations. Laryngoscope.

[29] Ghanem MA, Monroy A, Alizarde FS, Nicolau Y, Eavey RD (2006). Butterfly cartilage graft inlay tympanoplasty for large perforations. Laryngoscope.

[30] Akygit A, Karlidag T, Keles E, Kaygusuz I, Yalcın S, Polat C (2017). Endoscopic cartilage butterfly myringoplasty in children. Auris Nasus Larynx.

